# Supplementary material for: Evolution of imprinting via lineage-specific insertion of retroviral promoters
Source: Nat Commun. 2019 Dec 12;10:5674. doi: 10.1038/s41467-019-13662-9 (PMC6908575; doi:10.1038/s41467-019-13662-9)
Supplement: Supplementary file 3 — Description of Additional Supplementary Files [file 41467_2019_13662_MOESM3_ESM.pdf]

Description of Additional Supplementary Files

File Name: Supplementary Data 1
Description: Datasets used in this study

File Name: Supplementary Data 2
Description: Human and mouse igDMRs investigated in this study

File Name: Supplementary Data 3
Description: Oligonucleotide primers used in this study
