# Supplementary material for: Evolution of imprinting via lineage-specific insertion of retroviral promoters
Source: Nat Commun. 2019 Dec 12;10:5674. doi: 10.1038/s41467-019-13662-9 (PMC6908575; doi:10.1038/s41467-019-13662-9)
Supplement: Supplementary file 7 — Reporting Summary [file 41467_2019_13662_MOESM7_ESM.pdf]

nature research

Corresponding author(s): Lefebvre LL
Lorincz MC

Last updated by author(s): Nov 1, 2019

Reporting Summary

Nature Research wishes to improve the reproducibility of the work that we publish. This form provides structure for consistency and transparency in reporting. For further information on Nature Research policies, see Authors & Referees and the Editorial Policy Checklist.

Statistics

For all statistical analyses, confirm that the following items are present in the figure legend, table legend, main text, or Methods section.

n/a	Confirmed	
☐	☒	The exact sample size (*n*) for each experimental group/condition, given as a discrete number and unit of measurement
☐	☒	A statement on whether measurements were taken from distinct samples or whether the same sample was measured repeatedly
☐	☒	The statistical test(s) used AND whether they are one- or two-sided<br>*Only common tests should be described solely by name; describe more complex techniques in the Methods section.*
☐	☒	A description of all covariates tested
☒	☐	A description of any assumptions or corrections, such as tests of normality and adjustment for multiple comparisons
☐	☒	A full description of the statistical parameters including central tendency (e.g. means) or other basic estimates (e.g. regression coefficient) AND variation (e.g. standard deviation) or associated estimates of uncertainty (e.g. confidence intervals)
☒	☐	For null hypothesis testing, the test statistic (e.g. *F*, *t*, *r*) with confidence intervals, effect sizes, degrees of freedom and *P* value noted<br>*Give P values as exact values whenever suitable.*
☒	☐	For Bayesian analysis, information on the choice of priors and Markov chain Monte Carlo settings
☒	☐	For hierarchical and complex designs, identification of the appropriate level for tests and full reporting of outcomes
☒	☐	Estimates of effect sizes (e.g. Cohen's *d*, Pearson's *r*), indicating how they were calculated

Our web collection on statistics for biologists contains articles on many of the points above.

Software and code

Policy information about availability of computer code

Data collection	Data was collected using standard Illumina software for the NextSeq 500 and HiSeq 2500 platforms.
Data analysis	WGBS data: Bismark, SamTools, Bedtools, VisRSeq<br>RNA-seq data: Trimmomatic, STAR, Picard-Tools, Bedtools, VisRSeq<br>ChIP-seq data: Trimmomatic, Bowtie2, Picard-Tools, Samtools, Bedtools, VisRSeq

For manuscripts utilizing custom algorithms or software that are central to the research but not yet described in published literature, software must be made available to editors/reviewers. We strongly encourage code deposition in a community repository (e.g. GitHub). See the Nature Research guidelines for submitting code & software for further information.

Data

Policy information about availability of data

All manuscripts must include a data availability statement. This statement should provide the following information, where applicable:

- Accession codes, unique identifiers, or web links for publicly available datasets
- A list of figures that have associated raw data
- A description of any restrictions on data availability

RNA-polII CTD domain ChIP-seq datasets in mouse GVOs are available at Gene Expression Omnibus (GEO) under accession GSE126363 (Figs 3-4)
All published mouse, rat, human, macaque and chimpanzee datasets analyzed in this study are detailed in Supplementary Data 1.

Field-specific reporting

Please select the one below that is the best fit for your research. If you are not sure, read the appropriate sections before making your selection.

☒ Life sciences ☐ Behavioural & social sciences ☐ Ecological, evolutionary & environmental sciences

For a reference copy of the document with all sections, see nature.com/documents/nr-reporting-summary-flat.pdf

Life sciences study design

All studies must disclose on these points even when the disclosure is negative.

Sample size	No statistical method was used to pre-determine sample size.
Data exclusions	Duplicate reads or reads aligning to multiple genomic regions were excluded from analysis.
Replication	ChIP-seq data generated for this study was generated in duplicates.<br>Previously published datasets analyzed in this study are detailed in Supplementary Data 1. After confirming high correlation, duplicate and triplicate datasets were combined for final analysis and visualization.
Randomization	The experiments were not randomized.
Blinding	The investigators were not blinded to group allocation during sample collection or analysis (inter-species comparisons).

Reporting for specific materials, systems and methods

We require information from authors about some types of materials, experimental systems and methods used in many studies. Here, indicate whether each material, system or method listed is relevant to your study. If you are not sure if a list item applies to your research, read the appropriate section before selecting a response.

Materials & experimental systems

n/a	Involved in the study	
☐	☒	Antibodies
☒	☐	Eukaryotic cell lines
☒	☐	Palaeontology
☐	☒	Animals and other organisms
☒	☐	Human research participants
☒	☐	Clinical data

Methods

n/a	Involved in the study	
☐	☒	ChIP-seq
☒	☐	Flow cytometry
☒	☐	MRI-based neuroimaging

Antibodies

Antibodies used	Anti-RNA polII CTD domain mouse monoclonal antibody (Abcam ab817, clone 8WG16, lot GR3209198-1).<br>0.1 ug of antibody per ChIP reaction were used.
Validation	RNA polII signal was intersected with transcription initiation sites as identified by de novo transcriptome assembly of previously published mouse GVO RNA-seq datasets.

Animals and other organisms

Policy information about studies involving animals; ARRIVE guidelines recommended for reporting animal research

Laboratory animals	Mouse samples were collected from C57BL/6 females or C57BL/6 x Cast/Ei F1 animals. GVOs were isolated from 3-8 week-old females. Placentae, embryonic heads and Embryonic bodies were collected at E13.5 DPC. Ethical approval for mouse studies experiments were performed under certificates A15-0291, and A15-0181 from the UBC Animal Care Committee and complied with the national Canadian Council on Animal Care guidelines for the ethical care and use of experimental animals.<br><br>Three rhesus macaque (Macaca mulatta) placentas and their parental blood DNA as well as three chimpanzee (Pan troglodytes verus - provided from Kumamoto Zoo) placentas and their parental blood DNA were utilized for the primates study. All primates samples are obtained from outbred colonies. All primate experiments were approved by the animal experiment committee of Primate Research Institute (PRI) of Kyoto University (Approval No: 2018-004).
Wild animals	n/a

Field-collected samples	n/a
Ethics oversight	All mouse experiments were performed under certificates A15-0291, and A15-0181 from the UBC Animal Care Committee and complied with the national Canadian Council on Animal Care guidelines for the ethical care and use of experimental animals. All primate experiments were approved by the animal experiment committee of Primate Research Institute (PRI) of Kyoto University (Approval No: 2018-004).

Note that full information on the approval of the study protocol must also be provided in the manuscript.

ChIP-seq

Data deposition

☒ Confirm that both raw and final processed data have been deposited in a public database such as GEO.

☐ Confirm that you have deposited or provided access to graph files (e.g. BED files) for the called peaks.

Data access links<br>*May remain private before publication.*	RNA-polII CTD domain ChIP-seq datasets are available at Gene Expression Omnibus (GEO) under accession GSE126363, reviewer token: mvozocoylnsbxaf
Files in database submission	Raw fastq files (75 bp paired-end sequencing reads):<br>C57BL/6_oocyte_RNApolII_rep1.fastq.gz<br>C57BL/6_oocyte_RNApolII_rep2.fastq.gz<br>Processed bigwig files (MapQ>10, no duplicate reads, 1kb smoothing)<br>C57BL/6_oocyte_RNApolII_rep1.bigwig<br>C57BL/6_oocyte_RNApolII_rep2.bigwig
Genome browser session<br>(e.g. UCSC)	*Provide a link to an anonymized genome browser session for "Initial submission" and "Revised version" documents only, to enable peer review. Write "no longer applicable" for "Final submission" documents.*

Methodology

Replicates	ChIP-seq for RNA polII CTD domain was done in duplicate in C57BL/6J GVOs
Sequencing depth	rep1: 4,053,750 non-duplicate reads with MapQ >10<br>rep2: 5,416,085 non-duplicate reads with MapQ >10
Antibodies	Anti-RNA polII CTD domain mouse monoclonal antibody (Abcam ab817, clone 8WG16, lot GR3209198-1).<br>0.1 ug of antibody per ChIP reaction were used.
Peak calling parameters	Peak calling was not used for this study.
Data quality	RNA polII signal was intersected with transcription initiation sites as identified by de novo transcriptome assembly of previously published mouse GVO RNA-seq datasets.
Software	Bedtools, IGV and VisRSeq software. All are open access and readily available online.
